# Knowledge, attitude and behavior towards vaccinations among nursing- and health care students in Hesse. An observational study

**DOI:** 10.3205/zma001511

**Published:** 2021-11-15

**Authors:** Timm Tristan Berg, Sabine Wicker

**Affiliations:** 1 Plant Medical Services, Wetzlar, Germany; 2 University Hospital of Frankfurt, Company Medical Services, Frankfurt/M., Germany

**Keywords:** health care workers, vaccination, occupational health, occupational physician, health education, nurses

## Abstract

**Objective: **Work-related vaccinations are recommended for employees in nursing and health care professions due to their elevated risk of infection because of job-related exposure. These vaccinations prevent work-related infections, protect patients and help to maintain the medical infrastructure. Thorough training and imparting of knowledge about vaccinations and work-related infections are essential pillars of the vaccination decision and thus for achieving a protective immune status. The present study examines the knowledge, attitudes and behavior of nursing- and health care students in Hesse regarding work-related infections and vaccinations.

**Methods: **In spring 2018, seven nursing schools in Hesse took part in an anonymous survey study. A total of 690 surveys from students of various health care professions were included in the study. The content of the survey was based on the recommendations of the Standing Committee on Vaccination (STIKO), a literature review and sample questions from the World Health Organization (WHO) regarding “vaccine hesitancy”. Vaccination cards were also evaluated based on the STIKO recommendations concerning standard vaccinations and occupational vaccinations for health care workers.

**Results: **The risk of acquiring a work-related infection was estimated to be quite high over all years of training. Gaps in knowledge were particularly evident in the area of vaccinations. Only three quarter of those surveyed believed that the effectiveness of vaccinations has been proven, and nearly 30% believed that the doses of the drugs used in vaccines were dangerous. Over 80% of the students had never had an influenza vaccination documented in their vaccination card.

**Conclusions: **The knowledge about vaccinations imparted in the course of the education should be expanded. A special course on the subject of vaccinations and the immune system with practical elements could contribute to a better understanding of how vaccinations work and misunderstandings could be eliminated in early stages of the training through the dialog between the students and the teacher in the classroom and the occupational physician as part of preventive occupational health check-ups.

## Zusammenfassung

**Zielsetzung:** Beschäftige in der Krankenpflege und medizinischen Ausbildungsberufen sind berufsbedingt einer erhöhten Infektionsgefährdung ausgesetzt, daher bestehen für sie Empfehlungen für beruflich indizierte Impfungen. Diese dienen neben der Vermeidung arbeitsbedingter Infektionen auch dem Patientenschutz, sowie der Aufrechterhaltung der medizinischen Infrastruktur. Eine profunde Ausbildung und Wissensvermittlung über Impfungen und arbeitsbedingte Infektionen sind wichtige Grundlagen der Impfentscheidung und somit zur Erlangung eines schützenden Immunstatus. Die vorliegende Studie untersucht das Wissen, die Einstellung und das Verhalten von Auszubildenden in medizinischen Ausbildungsberufen in Hessen zu arbeitsbedingten Infektionen und Impfungen.

**Methodik:** Im Frühjahr 2018 nahmen sieben Pflegeschulen in Hessen an einer anonymen Fragebogenstudie teil. Insgesamt wurden 690 Fragebögen von Schülerinnen und Schülern verschiedener medizinischer Ausbildungsberufe in die Studie aufgenommen. Der Inhalt der Fragen orientierte sich an den Empfehlungen der Ständigen Impfkommission (STIKO), einer Literaturrecherche, sowie an den Beispielfragen der Weltgesundheitsorganisation (WHO) bezüglich „vaccine hesitancy“. Ebenso erfolgte die Auswertung von Impfpässen anhand der STIKO-Empfehlungen hinsichtlich Standardimpfungen, sowie den beruflich bedingten Impfungen für Beschäftigte im Gesundheitswesen.

**Ergebnisse: **Das Risiko an einer arbeitsbedingten Infektion zu erkranken wurde über alle Ausbildungsjahre hinweg als ziemlich groß eingeschätzt. Wissenslücken zeigten sich vor allem im Bereich der Impfungen. Nur knapp drei Viertel der Befragten hielt die Wirksamkeit von Impfung für belegt und fast 30% waren der Meinung, dass die Dosierungen der in Impfstoffen erhaltenen Arzneistoffe gefährlich sind. Über 80% der Schülerinnen und Schüler hatten noch niemals eine Influenza-Impfung erhalten.

**Schlussfolgerung: **Das im Rahmen der Ausbildung vermittelte Wissen über Impfungen sollte ausgebaut werden. Eine spezielle Unterrichtseinheit zum Thema Impfungen und Immunsystem mit praktischen Anteilen könnte zum besseren Verständnis über die Wirkungsweise von Impfungen beitragen und Missverständnisse könnten im Dialog der Schülerinnen und Schüler mit der Lehrkraft im Unterricht und dem Betriebsarzt im Rahmen der arbeitsmedizinischen Vorsorgen bereits in frühen Stadien der Ausbildung beseitigt werden.

## Introduction

Health care workers have a higher risk of infection due to their job responsibilities [1], [2]. Vaccinations are an integral part of the prevention of infectious diseases in employees and their patients [3], [4] and more importantly to protect those with preexisting conditions [5]. In addition, vaccinations are an important component in maintaining our medical infrastructure, as can be seen in the current SARS-CoV-2 pandemic.

The significance of health care workers in terms of role models and influencers with regard to the validation and acceptance of vaccinations [6], [7] also underscores the importance of good training especially in the area of vaccination medicine. They act as important information sources and recommendation providers for patients [8], and their relatives [9]. Yet there are significant vaccination gaps within the nursing profession. In an online survey in 2019/2020, 79.3% of physicians were vaccinated against influenza, in the same period the rate of nurses vaccinated was only 46.7% [10].

The assessment of one’s own risk of infection and getting sick, the knowledge about vaccinations and one's own attitude about the subject of vaccinations are important factors for the decision to get vaccinated [6], [11]. Employees in health care also belong to a group of the working population with the most work-related vaccination recommendations of the Standing Commission on Vaccination (STIKO) [12], [13] and are also often confronted with recurring vaccinations, for example the seasonal flu shot or new vaccinations (e.g. against COVID-19) throughout their careers. A current study about the opinion of hospital employees on the COVID-19 vaccination shows, however, that affiliation to the occupational group of nursing personnel was associated with significantly less willingness to get vaccinated [14]. It was therefore the goal of the current study to conduct a survey on the factors that influence the decision on vaccination in order to work out potential approaches for improving the stance on vaccination and the willingness to get vaccinated.

Occupational physicians for health care workers, assume a special role here, because their recommendations for occupationally indicated vaccinations depend on their knowledge about current vaccination recommendations, as well as their personal attitude towards vaccinations [15]. 

Health care workers are subject to mandatory occupational health check-ups due to their contact with biological materials as part of their job. The employer must arrange these occupational health check-ups for his employees in accordance with the Ordinance on Occupational Health Precautions (ArbMedVV) [https://www.gesetze-im-internet.de/arbmedvv/]. These health check-ups must be carried out by the employee before the start of work and at the latest 12 months thereafter

[https://www.baua.de/DE/Angebote/Rechtstexte-und-Technische-Regeln/Regelwerk/AMR/pdf/AMR-2-1.pdf?__blob=publicationFile]. This provides two intervention points for occupational physicians to inform students about vaccinations and infectious diseases during their education. In this regard, the present study investigated the extent to which students are informed about the vaccination services offered by their occupational physician and to what extent he/she is consulted as a source of information regarding vaccinations and infectious diseases.

With the inclusion of §23a in the Infection Protection Act in 2015 [https://www.gesetze-im-internet.de/ifsg/] and the 2020 Measles Protection and Vaccine Prevention Strengthening Act [16], health care workers are also subject to legal provisions. Last but not least as a building block for the elimination of measles in Germany [17]. This is a formal goal of German health care policy. According to the “National Action Plan 2015-2020 for the Elimination of Measles and Rubella in Germany”, adults born after 1970 and, above all, health care workers are among the population groups with a special need for action [18]. In addition, the World Health Organization (WHO) already declared vaccine hesitancy as one of the top ten risks to global health in 2019 [19]. 

## Methods

In spring 2018, seven nursing schools in Hesse took part in an anonymous survey study. All nursing schools contacted had agreed to participate. 476 health care and nursing students, 108 health care and pediatric nursing students, and 98 STA- (surgical technical assistant) or MTA- (medical technical assistant) students participated in the study, which was conducted between March and October 2018. The questionnaire consisted of 10 questions, plus a “knowledge test” with 9 statements about vaccinations to be assessed. 

The development of the questionnaire resulted from the vaccination recommendations of the STIKO [12], as well as sample questions of the WHO regarding “vaccine hesitancy” [20]. Based on the results of the literature review about important factors of a vaccination decision, the questions of the questionnaire were divided into three content-related topics: The “infections” section includes questions about assessing the level of knowledge on infectious diseases, preparation to avoid work-related infections and assessing the risk of contracting a work-related infection. The “vaccinations in general” section includes questions about the assessment of the level of knowledge regarding vaccinations, one’s own attitude towards vaccinations, as well as the conveying of knowledge about vaccinations during the training. The “vaccinations specifically” section includes questions about one’s own vaccination status and knowledge of work-related vaccination recommendations. Questions 9 and 10 of the questionnaire target the issue of vaccine availability and information. The knowledge test itself (question 11 of the questionnaire) was taken from a study by Zingg A. and Sigrist M. [21].

The questionnaire is located in attachment 1 .

The questionnaires were distributed either via the school administrators and class teachers, or they were handed out to the students at the beginning of a course on the topic “vaccinations and the immune system”, filled out anonymously on a voluntary basis, and the questions were answered mainly using multiple-choice answers. 

The given answer options were numerically scaled according to the number of possible answers related to the question, and the respective mean values (MV) with standard deviation (SD) were determined.

Statistical analysis of the data by analysis of variance or chi-square tests was performed using SPSS and Microsoft Excel. Groups were categorized according to type of training (health care and nursing, health care and pediatric nursing, STA/MTA), age, gender and year of training. 

Questionnaires in which questions about group membership were omitted or not answered clearly were removed from the respective group analysis. Questions that were omitted or not answered clearly were not included in the evaluation.

In addition, participating schools were offered a double lesson on infectious diseases and vaccinations. The contents of the lessons included an introduction to the theory of infections, on how the immune system works and vaccinations. Five of the seven participating schools accepted the offer of instruction, which was conducted by the study director himself.

Following the class, the students also had the opportunity to have their vaccination cards checked by the study director according to the current STIKO recommendations (standard vaccinations, as well as occupational vaccination recommendations for health care workers) – this opportunity was communicated to the students in advance via their respective class teacher. Each student received the card back along with an individual recommendation on outstanding vaccinations after collecting the anonymous data. Immunization status for the study was analyzed as descriptive statistics using Microsoft Excel. 

See attachment 2 for compilation of vaccination recommendations for evaluation.

## Results

Out of 722 questionnaires handed out, 690 questionnaires from students in all three years of vocational training were included in the study and statistically analyzed, which corresponds to a response rate of 95.6%. 

The individual groups showed the following distribution (see table 1 (Tab. 1)).

There were also 292 vaccination cards of students from all levels of training that were checked for completeness and evaluated.

### Evaluations of the questionnaires in the “infections” section

Knowledge of infectious diseases in general was rated as predominantly “good” to “average” (scale of one=very good to five=very poor). The mean value across all years of training was 2.66 (SD: 0.64). 93.3% of all respondents rated their knowledge between “average” and “very good” (643 of 689 responses). 

It was demonstrated that knowledge of infectious diseases increases with advanced training time. Thus, students in their third year of training estimated their knowledge significantly better than those in their first year of training (p=0.012). 

The students surveyed also indicated that they had been for the most part well informed regarding the prevention of work-related infections during the course of their training – the mean value across all years of training was 2.18 (SD: 0.84).

Here, too, an improvement became apparent in the course of the training. Thus, students in their first year of training still felt significantly less prepared than in the second and third years of training (p<0.001). 

A comparison of the three types of training with each other (health care and nursing, health care and pediatric nursing, and STA/MTA) showed no significant differences. 

In addition, students were asked to rate how likely they would think medical personnel are to contract a work-related infection.

Across all years of training, students indicated that they considered the likelihood ”fairly high”. The mean value here was 4.98 (SD: 1.26). 452 of 686 respondents (65.9%) rated the risk as “fairly high” or higher. 

**Figure 1:**
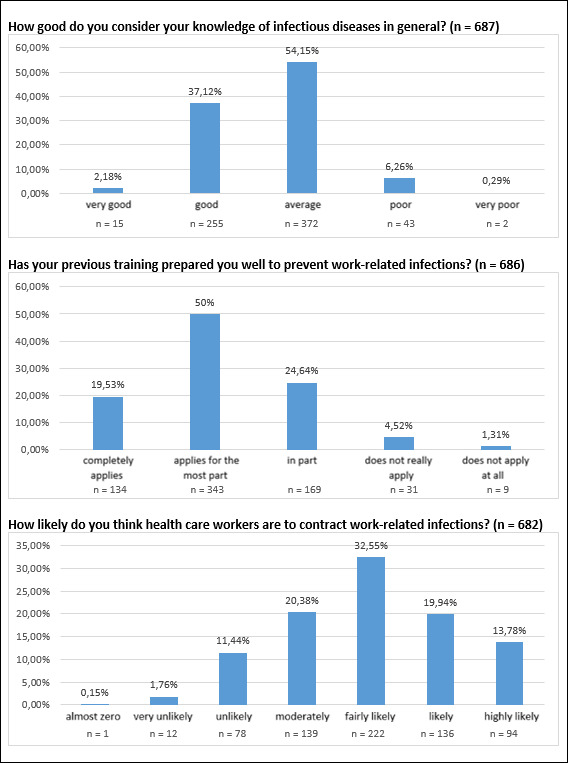
Frequency distribution of all evaluated answers in the “infections” section

The data for the individual questions in this area are shown in figure 1 (Fig. 1), as well as in table 2 (Tab. 2).

#### Evaluations of the questionnaires in the “vaccinations in general” section

Knowledge about vaccinations was rated as rather “average”. The mean value across all years of training was 2.84 (SD: 0.71). 85.2% of all respondents rated their knowledge between “average” and “very good” (588 of 690 responses).

In contrast to knowledge about infectious diseases, knowledge about vaccinations did not change significantly with increasing training time. It also showed that knowledge about vaccinations in general was rated significantly poorer than knowledge about infectious diseases in general (p<0.001). 

When asked about attitudes toward vaccination, there was a stable and positive attitude toward vaccination across all years of training. The mean value was 5.52 (SD: 1.21). 79.5% of respondents were “more pro vaccination” to “completely pro vaccination” (547 of 688 responses). 

The students surveyed also indicated that they had been for the most part “moderately” informed about vaccinations during the course of their training – the mean value across all years of training was 3.03 (SD: 0.87). Only 25% of all respondents reported being “very well” or “well” informed about vaccinations (172 of 688 responses).

Contrary to the assessment that knowledge about vaccinations did not significantly improve with increasing training time, this question showed that the more training time the students had already completed, the better they considered themselves informed about vaccinations – the third year of training showed a significant increase compared to the first and second year of training (p<0.001).

Evaluations by type of training showed that students in STA/MTA training felt themselves significantly less informed about vaccinations than students in health care and nursing or health care and pediatric nursing (p<0.001). 

**Figure 2:**
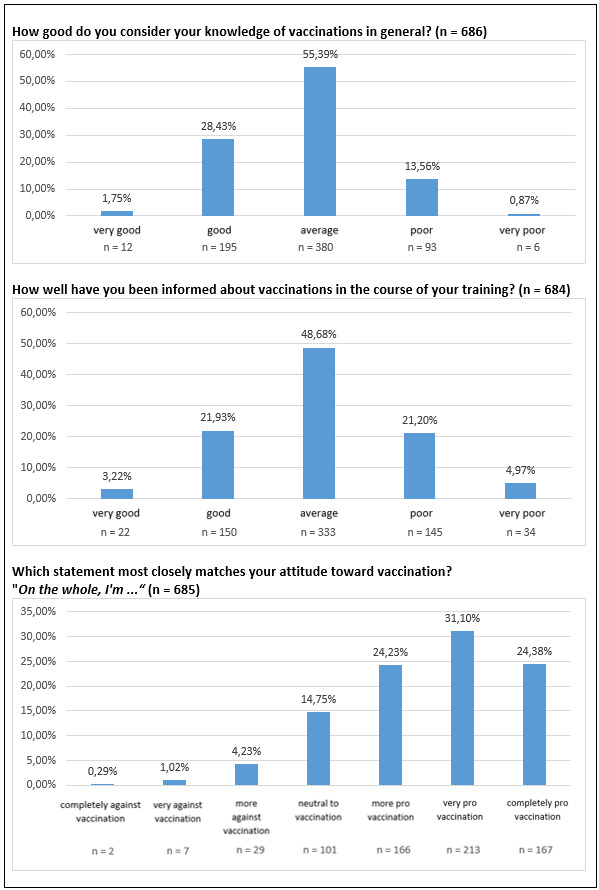
Frequency distribution of all evaluated responses in the area “vaccinations in general”

The data for the individual questions in this area are shown in figure 2 (Fig. 2), as well as in table 3 (Tab. 3).

#### Evaluations of the questionnaires in the “vaccinations specifically” section

Students were further asked about who they would contact if they needed information about vaccinations.

Consultation of the following sources, among others, increased as the training period progressed:

- occupational medical services (33.5% in the first year of training to 37.7% in the last year of training)
- primary physician (77.1% in the first year of training to 88.4% in the last year of training)
- textbooks (4.8% in the first year of training to 8.9% in the last year of training)
- official websites/forums (24.5% in the first year of training to 37% in the last year of training)

Consultation of the following source decreased as the training period progressed:

- experience of medical nonprofessionals (12.9% in the first year of training to 6.2% in the last year of training)

In addition, it was demonstrated that younger students (up to 25 years) were even more likely to seek advice from work colleagues (13.0% vs. 5.8%) or medical nonprofessionals (11.5% vs. 1.9%) than older students (over 25 years). They are also more likely to obtain information from textbooks (7.0% vs. 2.9%).

**Figure 3:**
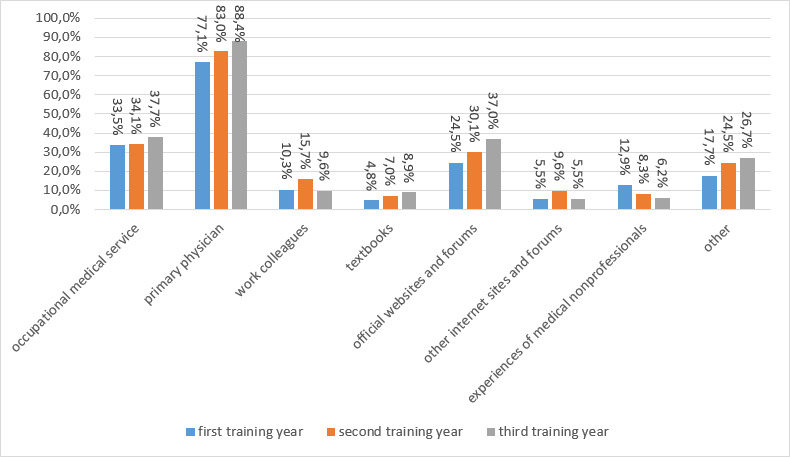
Sources consulted for vaccination questions, broken down by year of training. Selected answer as a percentage of respondents in the year of training.

The individual breakdown of responses can be found in figure 3 (Fig. 3).

In the first year of training, 18.9% of respondents were aware of the vaccination services offered by their occupational physician. In the third year of training, 26.6% of respondents stated that they were aware of the vaccination services offered by the occupational physician. Among STA/MTA students, the figure was just under 8%. 

At the same time, utilization of vaccinations by primary care physicians decreased from 25.5% in the first year of training to 17.8% in the last year of training.

The questions regarding vaccination recommendations showed that only about half of all students across all years of training stated that they had received all vaccinations recommended by the STIKO. Broken down by type of training, it was even just under 46% in the area of health care and nursing. As many as nearly ten percent reported not having received all of the recommended vaccinations, and about a quarter of all students said they thought they had received only some of them.

When evaluated by age, younger students were significantly more likely to report having received the recommended vaccinations than older students (p=0.039). 

In each year of training, only about half of all students indicated that they were aware that the STIKO recommends additional vaccinations for health care workers.

The data for the individual questions in this area are shown in table 4 (Tab. 4).

#### Evaluation of the knowledge test

**Figure 4:**
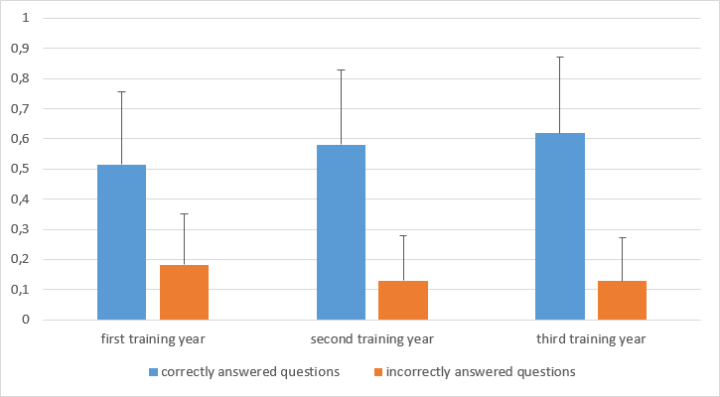
Evaluation of the knowledge test according to correctly and incorrectly answered questions. (Shown are both the mean values of the correctly answered questions and the mean values of the incorrectly answered questions with their respective standard deviations, broken down by training year.)

The knowledge test on the topic of vaccinations showed that the results of the second and third year of training were significantly better than those of the first year of training (p<0.01 and p<0.001) for both the correctly answered questions and the incorrectly answered questions (see figure 4 (Fig. 4)).

It was also found that the male students gave correct answers significantly more often than female students. On average, the male students had answered just under 64% of the questions correctly, while the female students had answered just under 55% (p<0.001).

**Figure 5:**
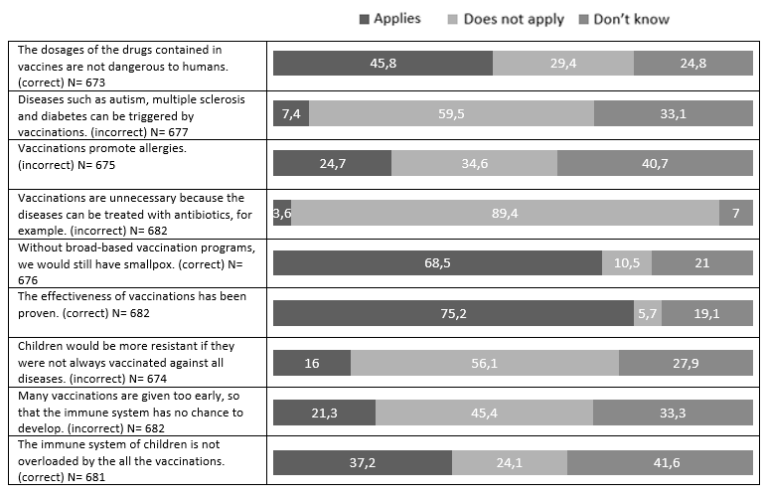
Answers of the knowledge test as a percentage of the answers given

Individual responses can be found in figure 5 (Fig. 5). 

#### Evaluations of the vaccination cards

Review of vaccination cards revealed high rates of complete basic immunization against tetanus, diphtheria, pertussis (between 95.2% and 100%), and polio (between 59.5% and 89.2%) in the 3 years of training. Current tetanus, diphtheria, pertussis vaccination ranged from 70.7% and 77.6%.

Two-time measles, mumps, rubella vaccination was present in 72.9%-89.5% of students.

Over the years of training, the proportion of students with complete hepatitis B and hepatitis A basic immunization also improved. Thus, 75% (n=53) of the students in the first year of training had complete basic immunization against hepatitis B, 85.4% (n=49) of the students in the second year of training, and 95.1% (n=41) in the final year of training. Complete basic immunization against hepatitis A was present in 18.8% of students in the first year of training (n=53), in 51% in the second year of training (n=49) and in 64.1% of students in the last year of training (n=41).

With regard to seasonal influenza vaccination, it was found that across all years of education, 82.5% of all students had never had an influenza vaccination documented on their vaccination card.

## Discussion

In the present study, the respondents' own risk of contracting a work-related infection was rated as fairly high irrespective of their year of training with nearly 66% of respondents rating the risk as “fairly high” or higher. The result is comparable to that of a similar question asked of medical students in a study by Petersen et al. [22]: Here, 68.6% of medical students had assessed their risk accordingly. This is an important finding, as the assessment of one’s own risk is an important reason for deciding whether to get vaccinated. For example, in a cross-sectional survey concerning nurses’ knowledge and risk perception of seasonal influenza by Zhang et al. [7], nurses with high perceived risk were more likely to get vaccinated against influenza than those with low perceived risk. In another review of influenza vaccination among health care workers in hospitals by Hollmeyer et al. [23], it was also shown that self-protection was often the most important reason for getting vaccinated.

One’s own attitude toward vaccination is also an important factor in the vaccination decision. For example, an older study among Danish physicians already showed that the average MMR vaccination rate was 85% when it was judged that MMR vaccination was “very helpful” – compared with 69% in practices that described MMR vaccination as “helpful” [24]. For occupational physicians, their own attitude also plays an important role in recommending vaccinations to health care workers under their care [15]. Knowledge about vaccines and their efficacy helps to increase health care workers’ confidence in vaccinations and thus their willingness to recommend vaccinations to others [25].

Our survey revealed a generally positive attitude towards vaccination, which was already evident in the first year of training. Nearly 80% of respondents had indicated a positive attitude toward vaccination. However, compared with the result of the study by Petersen et al. in which more than 90% of medical students had reported a positive attitude toward vaccination [22], there is still room for improvement. In addition, despite increased information about vaccination during training, vaccination attitudes had not significantly improved in our study. 

Misunderstanding or lack of knowledge about infections are often cited as barriers to get vaccinated [23]. In our study, both knowledge of infectious diseases and preparation to prevent work-related infections were better assessed as training progressed. On the one hand, the students seem to be aware of the risk of work-related infections and, on the other hand, they seem to have received a correspondingly good education in the area of infectious diseases.

Another important barrier to vaccination is the real or subjectively perceived lack of conveniently available vaccine [26]. Our survey showed that knowledge of the vaccinations offered by the occupational physician increased as training progressed, but despite this, only just over a quarter of students at the end of their training even knew which vaccinations were available to them by their occupational physician. At the same time, the use of primary care physicians for vaccinations declined from just over a quarter in the first year of training to just under 18% in the final year of training, although primary care physicians were increasingly cited as a potential source of information regarding vaccinations.

Here in particular, the involvement of occupational physicians in teaching about vaccinations and in passing on information about the vaccinations available (as well as in the context of the occupational health check-ups that take place anyway) and appropriately organized vaccination campaigns, could increase the willingness to be vaccinated. The combining of a practical approach with theoretical teaching (for example, by an occupational physician) can help improve instruction, as a recent study at the University Hospital of Frankfurt showed. Here, comparing a theoretical vaccination seminar to a practical one, the practical seminar was graded significantly better by medical students [27].

Knowledge about vaccinations in general is rated lower than knowledge about infections and does not increase over the course of education, although students reported being better informed about vaccinations over the course of their education. Students in STA/MTA training also perceived themselves to be significantly less informed about vaccinations than students in health and nursing or health and pediatric nursing. Joint courses on this topic, which is also relevant for students in the STA/MTA field, would be conceivable here.

Nevertheless, increased education about vaccinations seems to have had a positive effect over the years as both correct answers on the knowledge test increased over the course of training and incorrect answers decreased over the course of training. Regardless of this, however, gaps in knowledge were still apparent here as well. Almost a quarter of the respondents stated that they believe the effectiveness of vaccinations has not been proven (or they don‘t know) and almost 30% were of the opinion that the doses of drugs obtained in vaccines are dangerous. There is an urgent need for training in this area as doubts about the efficacy of vaccinations [28] and fear of side effects [7] are further important obstacles to being vaccinated.

Meanwhile, the reason male participants performed significantly better in the knowledge test remains speculative – at least the male participants showed a slightly higher willingness to make decisions: On average, only about 22% opted for the answer option “don’t know”; among the female participants, the figure was just under 29%.

Increased information about vaccinations also did not appear to result in students being better informed about current STIKO recommendations in their final year of training than at the beginning of their training. Only about half of all students across all years of training indicated that they thought they had received all of the vaccinations recommended by the STIKO, and likewise only about half of all students indicated that they were aware that the STIKO recommends any additional vaccinations at all for health care workers. This would mean that about half of all graduates would have incomplete vaccination protection at the end of the third year of training and thus shortly before starting their careers (assuming correct self-assessment) and would not know about vaccinations specifically recommended for health care workers.

However, review of the vaccination cards showed that the immunization status was better than the students’ assessment. Higher vaccination rates against hepatitis A and B were as students progressed in their training. 

Nevertheless, overall vaccination rates are in need of improvement. For example, nearly one-quarter of the students did not have current tetanus, diphtheria, and pertussis vaccinations. Vaccination against seasonal influenza also showed large gaps: Over 80% of the students had never had an influenza vaccination documented in their vaccination card. Moreover, no relevant change can be seen here in the course of training and there is still a clear need to level the knowledge gap in educational work, as studies show that nurses with a high level of knowledge about influenza vaccinations are more likely to be vaccinated against influenza than nurses with a low level of knowledge [7]. In addition, it is known from other studies that there is a positive relationship between one's own vaccination or willingness to get vaccinated and the recommendation for others to get vaccinated [7], [29], [30], [31]. 

### Limitations

As a limitation of the study, it should be noted that comparability in the group of the training year is limited, since at the time of the study in the respective nursing school not necessarily all students of a year cover the same topics in their classes and the curriculum varies depending on the type of training. Another limitation is the subjective self-assessment of the participants, which could also lead to answers which are socially desirable.

## Conclusions

The present study shows that students are aware of the risk of contracting a work-related infection during their careers. In this regard, the education seems to provide good information, especially with regard to the prevention of work-related infections. Vaccinations play a prominent role in this context. However, it is precisely in this area that there still seems to be a need to level the knowledge gap – also in school education. Thus, in addition to the existing courses on the topic of infectious diseases, the teaching curricula should also explicitly include the topic of vaccinations. Especially the dialog between teacher and students can help to close gaps in knowledge at an early stage and the integration of a practical reference can additionally increase the attractiveness of the curriculum.

Also, the topic of work-related infections will and should continue to be addressed and addressed in greater depth as part of occupational health check-ups. Information dissemination about vaccination supply should be intensified.

With the change in the STIKO vaccination recommendations for the occupationally indicated vaccinations against measles, mumps, rubella and varicella for health care workers in 2020 and the Measles Protection Act, occupational physicians' involvement with these vaccinations will also become even more important.

Many students did not have their vaccination cards with them at the time of the class (despite being informed of the offer to have their vaccination card checked anonymously). Here, a digital vaccination card could save searching in the future, as almost every young person has an appropriate app-enabled smartphone.

## Acknowledgements

The author thanks the participating nursing schools for their support.

## Competing interests

The authors declare that they have no competing interests. Prof. Wicker is vice-chair of the STIKO.

## Supplementary Material

Questionnaire

Evaluation based on the STIKO recommendations

## Figures and Tables

**Table 1:**
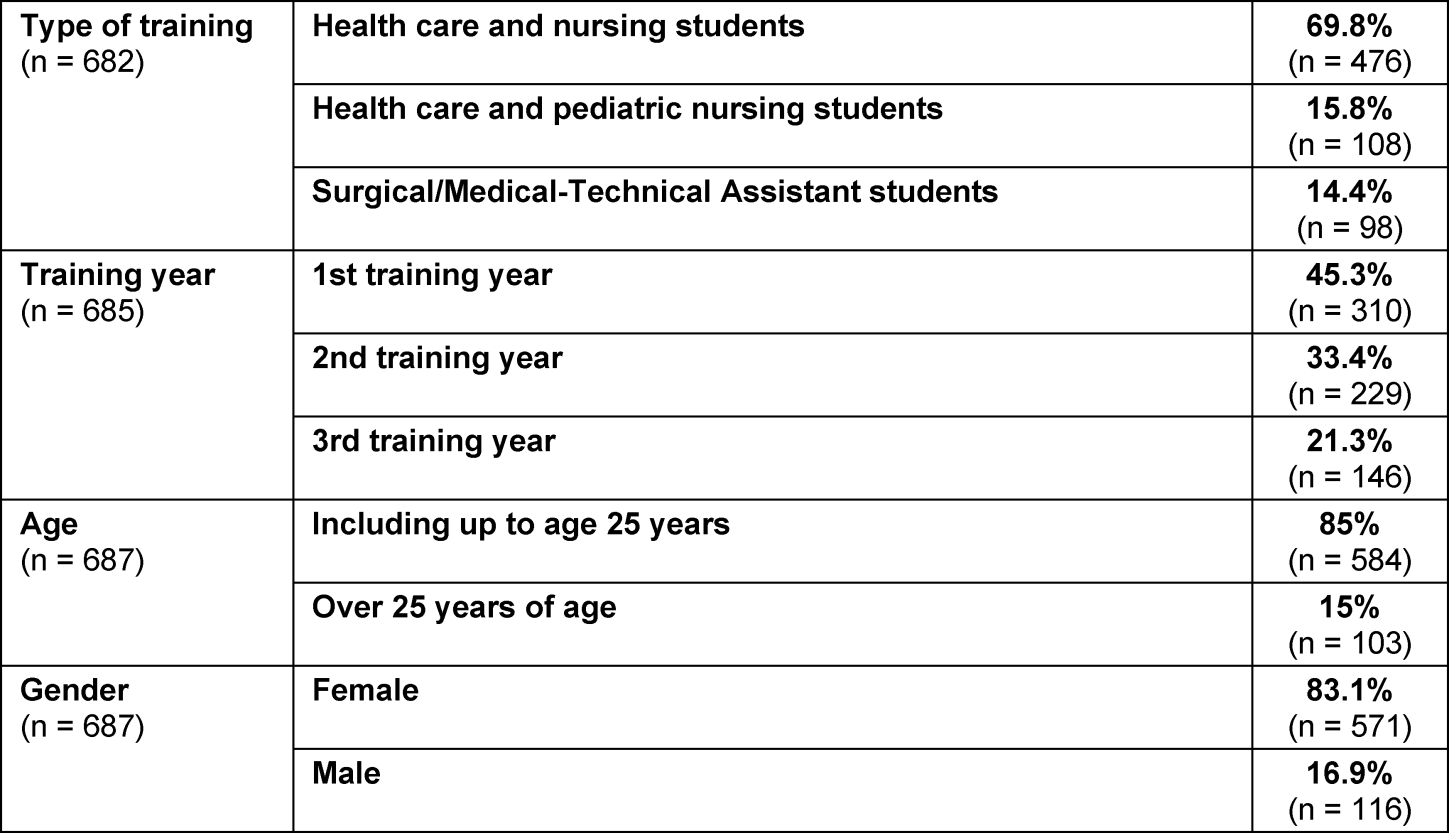
Division of the individual groups

**Table 2:**
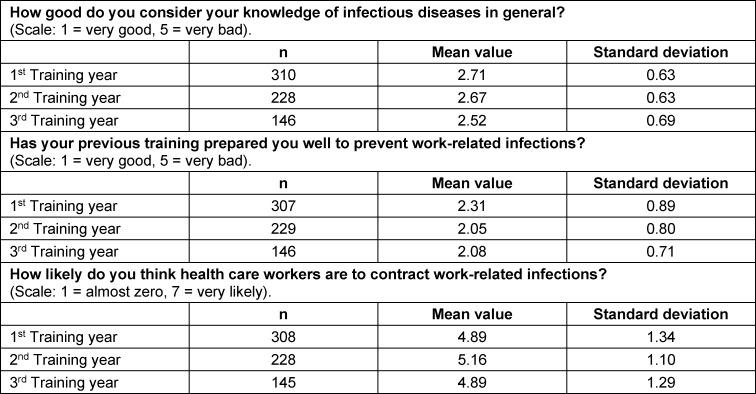
Responses of the topic “infections” broken down by year of training

**Table 3:**
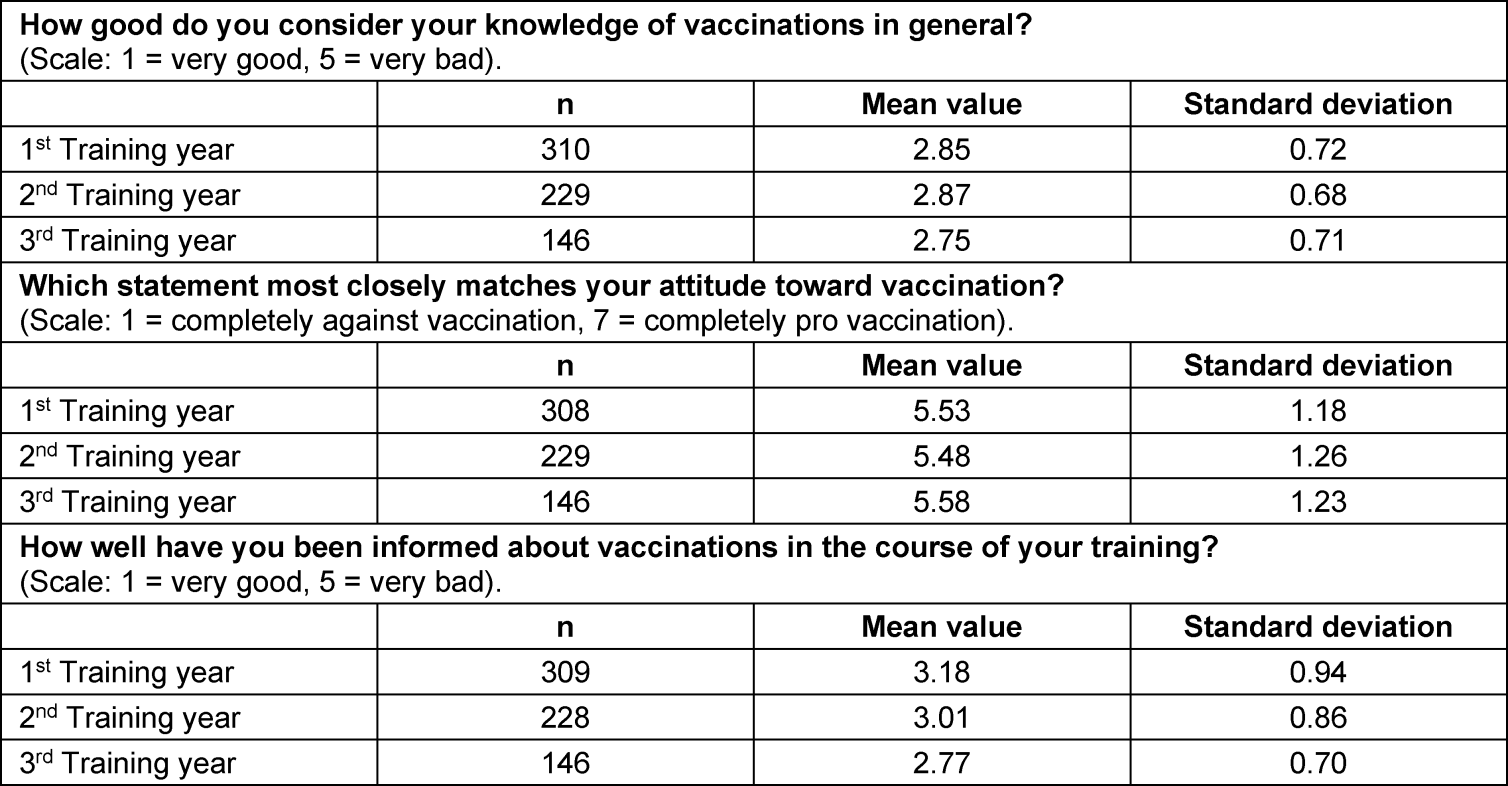
Responses of the topic “vaccinations in general” broken down by year of training

**Table 4:**
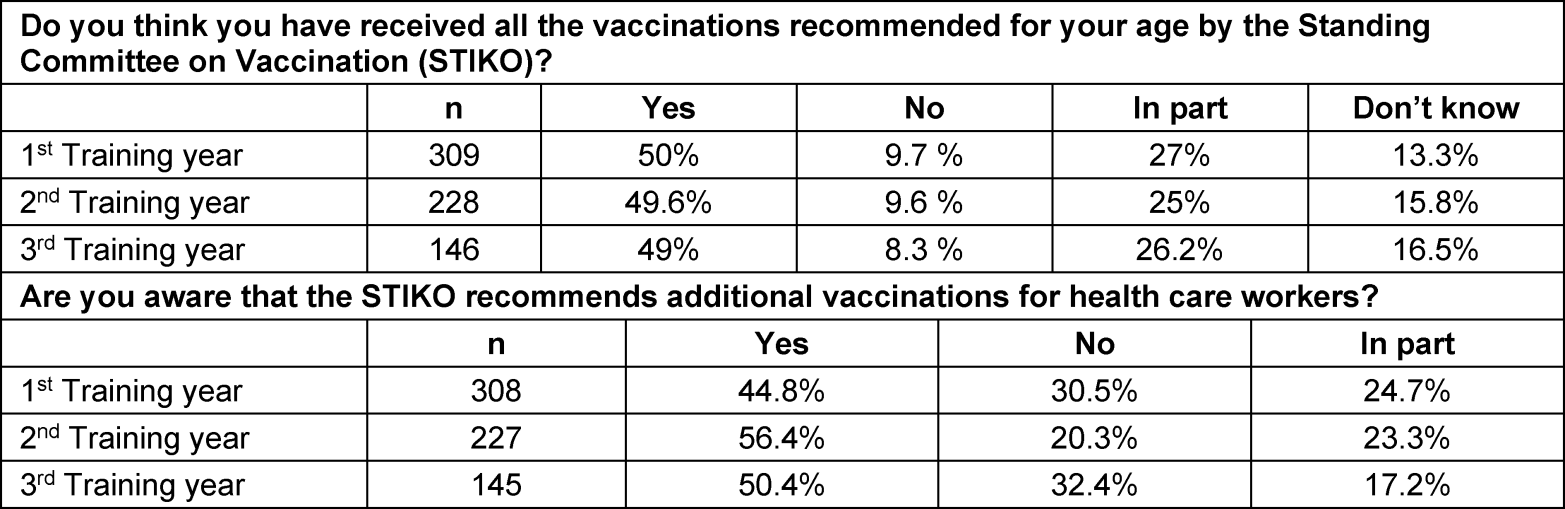
Responses of the topic “vaccinations specifically” broken down by year of training
