# Supplementary material for: Knowledge, attitude and behavior towards vaccinations among nursing- and health care students in Hesse. An observational study
Source: GMS J Med Educ. 2021 Nov 15;38(7):Doc115. doi: 10.3205/zma001511 (PMC8675377; doi:10.3205/zma001511)
Supplement: Questionnaire [file JME-38-7-115-s-001.pdf]

Attachment 1: Questionnaire

Questionnaire on the vaccination attitudes of students in health care professions

I am ❑ Health care and nursing student

❑ Health care and pediatric nursing student

❑ STA- / MTA- / RTA- / ATA student

In my ❑ 1st Training year ❑ 2nd Training year ❑ 3rd Training year

Gender ❑ female ❑ male

Age ❑ up to including 25 years of age ❑ above 25 years of age

Question 1 How good do you consider your knowledge of infectious diseases in general?

❑ Very good ❑ good ❑ average ❑ poor ❑ very poor

Question 2 How good do you consider your knowledge of vaccinations in general?

❑ Very good ❑ good ❑ average ❑ poor ❑ very poor

Question 3 Which statement most closely matches your attitude toward vaccination?

"On the whole, I'm ...“

❑	❑	❑	❑	❑	❑	❑
completely against vaccination	very against vaccination	more against vaccination	neutral to vaccination	more pro vaccination	very pro vaccination	completely pro vaccination

Question 4 Do you think you have received all the vaccinations recommended for your age by the Standing Committee on Vaccination (STIKO)?

❑ Yes ❑ No ❑ in part ❑ don’t know

Question 5 Are you aware that the STIKO recommends additional vaccinations for health care workers?

❑ Yes ❑ No ❑ in part

Question 6 How well have you been informed about vaccinations in the course of your training?

❑ Very well ❑ well ❑ average ❑ poor ❑ very poor

Question 7 Has your previous training prepared you well to prevent work-related infections?

❑	❑	❑	❑	❑
Completely applies	Applies for the most part	In part	Does not really apply	Does not apply at all

Attachment 1 to: Berg TT, Wicker S. *Knowledge, attitude and behavior towards vaccinations among nursing- and health care students in Hesse. An observational study*. GMS J Med Educ. 2021;38(7):Doc115. DOI: 10.3205/zma001511

Question 8 How likely do you think health care workers are to contract work-related infections?

❑ Almost zero ❑ very unlikely ❑ unlikely ❑ moderate ❑ fairly likely ❑ likely ❑ highly likely

Question 9 Do you know the vaccination offer of your occupational physician?

❑ Yes ❑ No ❑ in part ❑ go to my primary physician for vaccination

Question 10 Who do you contact if you need information about vaccinations?

❑ Company medical service ❑ primary physician

❑ Work colleagues ❑ Textbooks

❑ Official websites and forums (Robert Koch Institute, Paul Ehrlich Institute, authorities, World Health Organization, etc.)

❑ Other Internet sites and forums ❑ Experiences of medical nonprofessionals

Other____

Question 11 Please tick whether you think the statements are true or not:

	Applies	Does not apply	Don't know
The dosages of the drugs contained in vaccines are not dangerous to humans.	❑	❑	❑
Diseases such as autism, multiple sclerosis and diabetes can be triggered by vaccinations.	❑	❑	❑
Vaccinations promote allergies.	❑	❑	❑
Vaccinations are unnecessary because the diseases can be treated with antibiotics, for example.	❑	❑	❑
Without broad-based vaccination programs, we would still have smallpox.	❑	❑	❑
The effectiveness of vaccinations has been proven.	❑	❑	❑
Children would be more resistant if they were not always vaccinated against all diseases.	❑	❑	❑
Many vaccinations are given too early, so that the immune system has no chance to develop.	❑	❑	❑
The immune system of children is not overloaded by all of the vaccinations.	❑	❑	❑
