# Supplementary material for: Knowledge, attitude and behavior towards vaccinations among nursing- and health care students in Hesse. An observational study
Source: GMS J Med Educ. 2021 Nov 15;38(7):Doc115. doi: 10.3205/zma001511 (PMC8675377; doi:10.3205/zma001511)
Supplement: Evaluation based on the STIKO recommendations [file JME-38-7-115-s-002.pdf]

Attachment 2: Evaluation based on the STIKO recommendations

<u>Conceptualizations of the evaluations of the vaccination cards:</u>

"Tetanus, diphtheria, pertussis basic immunization complete" = received at least 3 vaccinations

"Current tetanus, diphtheria, pertussis vaccination" = vaccination not longer than 10 years ago

"Poliomyelitis basic immunization complete" = received at least 3 vaccinations

"Measles, mumps, rubella basic immunization complete" = received at least 2 vaccinations

"Hepatitis B basic immunization complete" = received at least 3 vaccinations

"Hepatitis A basic immunization complete" = received at least 2 vaccinations, or 3 x combination vaccination

"Influenza vaccination current" = a vaccination valid for the current season is available

Attachment 2 to: Berg TT, Wicker S. *Knowledge, attitude and behavior towards vaccinations among nursing- and health care students in Hesse. An observational study*. GMS J Med Educ. 2021;38(7):Doc115. DOI: 10.3205/zma001511
